# The burden attributable to headache disorders in children and adolescents in Lithuania: estimates from a national schools-based study

**DOI:** 10.1186/s10194-021-01237-3

**Published:** 2021-04-13

**Authors:** Diana Genc, Nerija Vaičienė-Magistris, Apolinaras Zaborskis, Tayyar Şaşmaz, Aylin Yeniocak Tunç, Derya Uluduz, Christian Wöber, Çiçek Wöber-Bingöl, Timothy J. Steiner

**Affiliations:** 1 Department of Neurology, Faculty of Medicine, Lithuanian University of Health Sciences, Kaunas, Lithuania; 2 Department of Preventive Medicine and Health Research Institute, Faculty of Public Health, Lithuanian University of Health Sciences, Kaunas, Lithuania; 3 Department of Public Health, Mersin University School of Medicine, Mersin, Turkey; 4 Neurology Department, Cerrahpaşa School of Medicine, Istanbul University, Istanbul, Turkey; 5 Department of Neurology, Medical University of Vienna, Vienna, Austria; 6 Dr Gönül Bingöl-Dr Muammer Bingöl Çocuk ve Ergen Başağrısı Derneği – Society for Headache in Children and Adolescents, Suadiye, Istanbul, Turkey; 7 Department of Neuromedicine and Movement Science, Norwegian University of Science and Technology, Edvard Griegs gate, Trondheim, Norway; 8 Division of Brain Sciences, Imperial College London, London, UK

**Keywords:** **C**hild and adolescent headache, Migraine, Tension-type headache, Medication-overuse headache, Undifferentiated headache, Epidemiology, Burden of disease, Schools-based study, Lithuania, Global campaign against headache

## Abstract

**Background:**

We recently showed headache to be common in children (aged 7–11 years) and adolescents (aged 12–17) in Lithuania. Here we provide evidence from the same study of the headache-attributable burden.

**Methods:**

Following the generic protocol for *Lifting The Burden*’s global schools-based study, this cross-sectional survey administered self-completed structured questionnaires to pupils within classes in 24 nationally representative schools selected from seven regions of the country. Headache diagnostic questions were based on ICHD-3 beta criteria but for the inclusion of undifferentiated headache (UdH; defined as mild headache with usual duration < 1 h). Burden enquiry was conducted in multiple domains.

**Results:**

Questionnaires were completed by 2505 pupils (1382 children, 1123 adolescents; participating proportion 67.4%), of whom 1858 reported headache in the preceding year, with mean frequency (±SD) of 3.7 ± 4.5 days/4 weeks and mean duration of 1.6 ± 1.9 h. Mean proportion of time in ictal state, estimated from these, was 0.9% (migraine 1.5%, probable medication-overuse headache [pMOH] 10.9%). Mean intensity on a scale of 1–3 was 1.6 ± 0.6 (mild-to-moderate). Symptomatic medication was consumed on 1.5 ± 2.8 days/4 weeks. Lost school time was 0.5 ± 1.5 days/4 weeks (migraine 0.7 ± 1.5, pMOH 5.0 ± 7.8) based on recall, but about 50% higher for migraine according to actual absences recorded in association with reported headache on the preceding day. More days were reported with limited activity (overall 1.2 ± 2.4, migraine 1.5 ± 2.2, pMOH 8.4 ± 8.5) than lost from school. One in 30 parents (3.3%) missed work at least once in 4 weeks because of their son’s or daughter’s headache. Emotional impact and quality-of-life scores generally reflected other measures of burden, with pMOH causing greatest detriments, followed by migraine and tension-type headache, and UdH least. Burdens were greater in adolescents than children as UdH differentiated into adult headache types.

**Conclusions:**

Headache in children and adolescents in Lithuania is mostly associated with modest symptom burden. However, the consequential burdens, in particular lost school days, are far from negligible for migraine (which is prevalent) and very heavy for pMOH (which, while uncommon in children, becomes four-fold more prevalent in adolescents). These findings are of importance to both health and educational policies in Lithuania.

## Introduction

In our recently published prevalence study, we showed headache to be common in children (aged 7–11 years) and adolescents (aged 12–17) in Lithuania [1], as we have with similar schools-based methodology in Turkey [2], Austria [3], Ethiopia [4] and Mongolia [5]. And we confirmed, again, the need to recognise and include undifferentiated headache (UdH; defined as mild headache with usual duration < 1 h) if accounts of headache in these age groups are to be complete.

The prevalence study was part of a global enquiry into child and adolescent headache [6], an ongoing programme conducted by *Lifting The Burden* (LTB) [7–10] in its endeavour within the Global Campaign against Headache [7–9] to measure the scale and scope of headache-attributed burden worldwide. From the public-health perspective, while disease prevalence determines the magnitude of disease-attributed burden in a population, it is not of itself very informative. In this paper, we present estimates of burden based on the prevalence findings [1] together with evidence of burden collected directly and contemporaneously from the same sample. These estimates will inform both the Global Campaign and the Global Burden of Disease (GBD) study [11], but, of more immediate importance, they will inform local health and educational policies.

Institutional schooling is mandatory for 10 years in Lithuania [1, 12]. Hence, schools-based sampling, according to the generic protocol, has high methodological validity in this country.

## Methods

The methodology of this cross-sectional survey, using self-completed structured questionnaires administered to pupils in nationally representative schools, is already published [1, 13], as is the generic protocol on which it is based [6]. It will only be summarised here.

### Ethics and approvals

The study was approved by Kaunas Regional Committee of Bioethics (BE-2-7, 26-01-2016) and authorised by the relevant Regional Education Authorities. School managers and teachers agreed to their schools’ participation. Each participating child or adolescent, along with a parent, gave consent prior to inclusion.

Data were collected anonymously, and data protection legislation was complied with.

### Sampling and recruitment

We conducted the survey in Spring 2016 in 24 randomly selected schools located in seven regions of Lithuania purposively chosen to be representative of the country’s limited diversities. In each school, we included all pupils in each class across the age ranges 7–11 years and/or 12–17 years unless they or their parents had not consented. Following published recommendations [6], we aimed for *N* = 2200 evaluable participants (200 of each age 7–17 years).

### Survey instruments

The child and adolescent versions of LTB’s Headache-Attributed Restriction, Disability, Social Handicap and Impaired Participation (HARDSHIP) structured questionnaire [6] were administered to the pupils in class, and completed under supervision. Teachers received additional questionnaires enquiring into relevant school variables.

Diagnoses were made algorithmically during analysis according to HARDSHIP methodology [14]. ICHD-3 beta criteria [15] were followed, except for UdH, which was diagnosed by the two characteristics of mild intensity and short duration (< 1 h), whether or not migraine-like features were present [1, 2].

Burden enquiry included symptom burden (frequency of headache, and its usual duration and intensity during episodes), symptomatic medication intake (frequency), lost time from schooling and other activities as well as lost parental work time (using adaptations of the Headache-Attributed Lost Time (HALT) index [16]), and selected (headache-relevant) questions from KINDL® [17] addressing concentration, emotional impact and quality of life (QoL) [6]). Both these question sets are modules within child and adolescent HARDSHIP [6]. A validation study of the Lithuanian version of the QoL scale demonstrated conformity with the concepts of the original instrument and an acceptable internal consistency (Cronbach’s alpha: 0.749) [13]. QoL enquiry was addressed to all participants, with or without headache. Time frames of enquiry were the preceding 4 weeks, 1 week and 1 day (headache yesterday [HY]).

We transferred data into SPSS using independent double data-entry, reconciling discrepancies by reviewing source data.

### Analysis

Analyses were performed using the SPSS statistical package (version 21; IBM SPSS Inc., Chicago, IL, 2012).

We expressed reported headache frequency [F] and medication intake in terms of days in the preceding 4 weeks. We expressed headache intensity on a scale of 1–3 (equating to the reported categories “mild”, “moderate” and “severe”). Duration of episodes [D] was reported in hours as < 1, 1–2, 2–4 or > 4; for analysis we expressed these according to their mid-points (0.5, 1.5, 3 and 8 respectively, assuming a range of 4–12 h for the last), judging these approximations to be no less reliable than participants’ estimates of duration. From F and D we calculated proportion of time in ictal state (pTIS) according to the formula (pTIS = F/28*D/24), and expressed it as a percentage. We calculated disability at individual level (using this term in the sense imputed in GBD) as the product of pTIS and disability weight (DW) attributed in GBD 2013 [18] to the ictal state of each headache type. We estimated the population-level disability as the product of mean individual disability and prevalence. We expressed lost school time because of headache in days in the preceding 4 weeks, counting not going to school as a whole day and leaving school early as a half-day. We separately counted days of limited activity (defined as “could not do things you wanted to because of your headaches”). We counted parents reported to have lost time from their own work (regardless of quantity) during the same period. We counted participants reporting HY and the intensity of it, and those (as proportions) reporting a lost school day because of HY. We estimated predicted values of lost school yesterday as the product of number affected by headache and mean reported days (divided by 20) lost per pupil over the preceding 4 weeks. Responses to questions addressing concentration, emotional impact and QoL were all on a 4-point Likert scale (“never”, “sometimes”, “often”, “always”) [6], also referring to the preceding 4 weeks. We scored these 0–3, and summed them to generate emotional impact score (including concentration; potential range 0–18, high being adverse) and QoL score (0–36, low being adverse).

We expressed proportions as %, with 95% confidence intervals [CIs], and used chi-squared tests for comparisons. We treated all other variables as continuous, and used descriptive statistics (means and standard deviations [SDs]). For comparisons of continuous data, we used ANOVA when these were distributed normally, and otherwise Kruskal-Wallis test with post hoc Dunn test. We calculated relative risk (RR) of consequential outcomes.

We considered *p* < 0.05 to be significant.

## Results

Of the 3714 potential participants, 2505 completed the questionnaires (non-participating proportion 32.6%, due mostly to withheld or unavailable parental consent). Of these, 1169 (46.7%) were male and 1336 (53.3%) female; 1382 (55.2%) were children and 1123 (44.8%) adolescents; overall mean age was 11.5 ± 3.2 years (median 11) [1]. Gender- and age-adjusted 1-year prevalence of any headache was 76.6%, of migraine 21.4%, of tension-type headache (TTH) 25.6%, of UdH 24.0%, of all headache on ≥15 days/month 3.9% and of probable medication-overuse headache (pMOH) 0.8%. There were 42 undiagnosed headaches (1.7%). These data have been reported previously [1].

### Symptom burden

#### Headache frequency, duration and intensity

Table 1 shows these reported variables for headache overall and by headache type, along with estimated pTIS.

**Table 1:** Symptom burden overall and by headache type (*N* = 2505)

Headache type	n	Mean frequency (days/4 weeks)	Mean duration (hours)	Estimated proportion of time in ictal state (%)	Mean intensity (scale 1–3)^a^
All headache (all ages)	1858	3.7 ± 4.5	1.6 ± 1.9	0.9	1.6 ± 0.6
ages 7–11 y	866	2.8 ± 3.7	1.4 ± 1.8	0.6	1.6 ± 0.6
ages 12–17 y	992	4.5 ± 5.0	1.8 ± 2.0	1.2	1.6 ± 0.6
Migraine	519	3.9 ± 3.3	2.5 ± 2.5	1.5	2.0 ± 0.5
Tension-type headache	605	3.2 ± 3.0	1.8 ± 1.7	0.9	1.7 ± 0.5
Probable medication-overuse headache	18	19.7 ± 4.1	3.7 ± 3.2	10.9	2.2 ± 0.5
Other headache on ≥15 days/month	73	18.4 ± 4.3	2.5 ± 2.4	6.9	2.0 ± 0.6
Undifferentiated headache	601	1.8 ± 2.2	0.5 ± < 0.1	0.1	1.0 ± < 0.1

Means are presented ± SDs. ^a^Equating 1–3 to the reported categories “mild”, “moderate” and “severe”, and treating as continuous data

Frequency of headache overall was 3.7 days/4 weeks, trending upwards with age (Table 1). Headache on ≥15 days/month (including pMOH) was, of course, more frequent than the episodic headaches but, among the latter, UdH was less frequent (*p* < 0.001) than migraine or TTH. Headache episodes were relatively short-lasting; the overall mean of 1.6 h, also trending upwards with age, was brought down by UdH (Table 1), which, as expected, was of shorter duration (*p* < 0.001) than all other headache types. Because overall duration was short, overall mean pTIS was low (0.9%), and in children (0.6%) half that in adolescents (1.2%). For the episodic headaches, pTIS ranged from only 0.1% for UdH to 1.5% for migraine, but it reached 10.9% for pMOH (Table 1).

Mean headache intensity was 1.6 ± 0.6 overall (mild-to-moderate), with no trend across ages. Intensity was greater for migraine than TTH (*p* < 0.001), whereas UdH, again as expected, was rated less intense than all other headache types (all *p* < 0.001) (Table 1).

#### Medication use

Symptomatic medication intake in the preceding week and 4 weeks is shown in Table 2, with reported frequencies generally consistent in the responses to the two enquiries. This, also, trended upwards with age, an observation apparent in the 4-week enquiry, although frequency averaged less than twice a month overall, even among adolescents. Medication was taken more often for migraine than for TTH or UdH (both *p* < 0.001) and, of course, much more frequently for pMOH than for all other headache types (all *p* < 0.001) (Table 2).

**Table 2:** Symptomatic medication use (frequency) overall and by headache type (*N* = 2505)

Headache type	n	Preceding week	Preceding 4 weeks
		days (mean ± SD)	
All headache (all ages)	1858	0.5 ± 1.0	1.5 ± 2.8
ages 7–11 y	866	0.5 ± 1.0	1.1 ± 2.3
ages 12–17 y	992	0.5 ± 1.0	1.8 ± 3.1
Migraine	519	0.8 ± 1.2	2.0 ± 2.5
Tension-type headache	605	0.4 ± 0.9	1.3 ± 2.0
Probable medication-overuse headache	18	3.9 ± 1.7	18.2 ± 4.2
Other headache on ≥15 days/month	73	1.1 ± 1.4	3.9 ± 4.2
Undifferentiated headache	601	0.2 ± 0.6	0.5 ± 1.1

Comparison with Table 1 reveals that medication days were fewer than headache days, although negligibly so for pMOH,

#### Disability

DWs have been allocated by GBD 2013 only to the ictal states of migraine, TTH and pMOH [18]. These are shown in Table 3, along with disability, in the sense used by GBD, calculated accordingly at individual and population levels. The values in Table 3 are estimates of lost health, such that 0% would imply no detriment to health due to the headache type and 100% would indicate total loss, a state valued as no better than being dead [18]. At individual level, pMOH was the most disabling headache type by a substantial margin (almost 4-fold, compared with migraine), but, with prevalence factored in, migraine was far ahead at population level.

**Table 3:** Disability by headache type (*N* = 2505)

Headache type	n	Disability weight (from [18])	Disability (%)	
			Individual (mean)	Population
Migraine	519	0.441	0.64	0.13
Tension-type headache	605	0.037	0.03	0.008
Probable medication-overuse headache	18	0.223	2.4	0.018

#### Lost productive time

Table 4 shows these data, together with headache frequencies for comparison. Lost school time per affected pupil because of any headache averaged 0.5 days/4 weeks (*ie*, 0.5 days in 20 [2.5%], assuming a 5-day week). This, again, showed an upward trend with age (Table 4). Lost school time was greater for migraine than for TTH (*p* < 0.01) or UdH (*p* < 0.001), while pMOH cost those affected one quarter (5/20) of their school days.

**Table 4:** Lost productive time because of headache in the preceding 4 weeks, overall and by headache type (*N* = 2505)

Headache type	n	Headache frequency	Lost school time^a^	Limited activity^a^	Parent missed work^a^
		days/4 weeks/affected pupil (mean ± SD)			n (%)
All headache (all ages)	1858	3.7 ± 4.5	0.5 ± 1.5	1.2 ± 2.4	61 (3.3)
ages 6–11 y	866	2.8 ± 3.7	0.4 ± 1.5	0.9 ± 2.1	53 (6.1)
ages 12–17 y	992	4.5 ± 5.0	0.6 ± 1.6	1.3 ± 2.7	8 (0.8)
Migraine	519	3.9 ± 3.3	0.7 ± 1.5	1.5 ± 2.2	36 (6.9)
Tension-type headache	605	3.2 ± 3.0	0.4 ± 1.0	1.0 ± 1.8	12 (2.0)
Probable medication-overuse headache	18	19.7 ± 4.1	5.0 ± 7.8	8.4 ± 8.5	3 (16.7)
Other headache on ≥15 days/month	73	18.4 ± 4.3	1.2 ± 2.5	4.6 ± 5.9	2 (2.4)
Undifferentiated headache	601	1.8 ± 2.2	0.2 ± 0.8	0.7 ± 1.5	6 (1.0)

^a^See text for explanation

For all headaches, a greater proportion of days were reported with limited activity than lost from school, even allowing for a denominator for the former of 28 rather than 20. While days of limited activity were clearly related to headache frequency, with ratios of the order of 1:3 for the episodic headaches, pMOH again stood out, affecting almost 1 day in every three (8.4/28).

In Table 5 are data relating to HY. Participants with migraine and reporting HY were more likely to have lost school time yesterday than those with other headache types and reporting HY, but numbers were too few for significance (RR = 1.5 [0.9–2.3]; *p* = 0.1163). They were also too few to relate likelihood of having missed school to reported intensity of HY.

**Table 5:** Headache yesterday (HY) and lost school time yesterday, overall and by headache type (*N* = 2505)

Headache type	n	Headache yesterday			
		Proportion reporting HY n (%)	Intensity of HY scale 1–3^a^ (mean ± SD)	Missed school day yesterday n (% of those reporting HY)	
				Actual	Predicted
All headache	1858	438 (23.6)	1.6 ± 0.7	62 (14.2)	46 (10.6)
Migraine	519	160 (30.8)	1.8 ± 0.7	28 (17.5)	18 (11.4)
Tension-type headache	605	127 (21.0)	1.5 ± 0.6	16 (12.6)	12 (9.5)
Probable medication-overuse headache	18	13 (72.2)	2.2 ± 0.7	1 (7.7)	4 (30.8)
Other headache on ≥15 days/month	73	58 (79.5)	1.8 ± 0.6	4 (6.9)	4 (6.9)
Undifferentiated headache	601	68 (11.3)	1.1 ± 0.3	11 (16.2)	6 (8.8)

^a^Equating 1–3 to the reported categories “mild”, “moderate” and “severe”, and treating as continuous data

Table 5 compares actual lost school yesterday with predictions based on the 4-week (recalled) data of Table 4 (column 4). Thus, for example, the prediction for all headache was (0.5/20)*1858 = 46. For the episodic headaches, actual values were always somewhat higher than predicted values. For pMOH and other headache on ≥15 days/month, numbers were too small to support analysis.

One in 30 parents (3.3%) missed work at least once in 4 weeks because of their son’s or daughter’s headache, but this was clearly age-related, and in the opposite direction to other variables trending with age, so that parents of children were at much higher risk than those of adolescents (RR = 7.6 [95% CI: 3.6–15.9]; *p* < 0.0001). These data are in Table 4. Risk also varied with headache type, rising two-fold (6.9%) for migraine (*p* < 0.001) and five-fold, to one in six (16.7%), for pMOH, although numbers for the latter were small. Taking UdH as reference, the RR for a parent missing work ranged from 2.0 (0.75–5.3; *p* = 0.1669) for TTH through 6.9 (3.0–16.4; *p* < 0.001) for migraine to 16.7 (4.5–61.5; *p* < 0.001) for pMOH.

#### Emotional impact and quality of life (QoL)

Emotional impact scores, with a median value of 5.0, were not normally distributed but, judged nonetheless by means, UdH had least emotional impact (*p* < 0.001 vs each other headache type), followed by TTH (Table 6). Migraine, pMOH and other headache on ≥15 days/month were not significantly different from each other, but all had greater impact (*p* < 0.001) than TTH.

**Table 6:** Emotional impact and quality of life, overall and by headache type (*N* = 2505)

Headache type	n	Emotional impact score^a^ scale 0–18^a^ (mean ± SD)	Quality-of-life score^a^ scale 0–36^a^ (mean ± SD)
No headache	647	–	25.8 ± 4.7
All headache	1858	5.2 ± 3.0	23.7 ± 5.2
Migraine	519	6.6 ± 3.0	21.9 ± 5.5
Tension-type headache	605	5.0 ± 2.7	24.0 ± 4.8
Probable medication-overuse headache	18	8.9 ± 3.2	19.2 ± 7.3
Other headache on ≥15 days/month	73	7.1 ± 2.9	20.8 ± 5.2
Undifferentiated headache	601	3.9 ± 2.5	25.4 ± 4.5

^a^See text for explanation

QoL scores, with a median value of 25.0, were also not normally distributed. Again judged by means, UdH had no impact on QoL when compared with no headache, unlike all other headache types (Table 6). Numerically, pMOH had greatest impact but, because of a wide SD, differed significantly only from UdH (*p* < 0.01) and no headache (*p* < 0.001). Migraine had more impact than TTH (*p* < 0.001), which itself was associated with a lower QoL score than UdH or no headache (both *p* < 0.001) (Table 6).

## Discussion

We previously reported that headache was very common among children and adolescents in Lithuania, with a 1-year prevalence of 76.6%, very roughly one third each of migraine (21.4%), TTH (25.6%) and UdH (24.0%) [1]. Disorders characterised by headache on ≥15 days/month were far from rare (3.9%), although fewer than a quarter of these were pMOH (0.8%) [1]. One in six participants (17.5%) in this survey recorded HY, effectively the 1-day prevalence of headache [1]. Inevitably, as we show here, there were associated burdens, measurably expressed in almost all domains of enquiry. In summary, mean headache frequency was 3.7 days/4 weeks, generating an estimated mean pTIS of 0.9% (1.5% for migraine, 10.9% for pMOH) and estimated disabilities (loss of healthy life) of 0.64% for migraine and 2.4% for pMOH; mean intensity was 1.6 (mild-to-moderate); symptomatic medication was consumed on a mean of 1.5 days/4 weeks. Consequentially, lost school time averaged 0.5 days/4 weeks overall. For participants with migraine, school was missed on 0.7 days/4 weeks according to recall over that period, but actual absences recorded in association with HY were about 50% higher. Those with pMOH reported missing school on 5.0 days/4 weeks. More days were reported with limited activity than were lost from school (notably, about one in every three for pMOH). One in 30 parents (3.3%) missed work at least once in 4 weeks because of their son’s or daughter’s headache. Emotional impact and QoL scores generally reflected other measures of burden.

The symptomatic burden of child and adolescent headache does not, from these findings, generally appear to be heavy, with a mean pTIS of < 1% and only mild-to-moderate headache on average. Estimates of lost health based on pTIS and DWs from GBD 2013 [18] ranged from a very low 0.03% for TTH through 0.64% for migraine to 2.4% for pMOH. To be clear what these mean, 1.0% would equate to one entire year of healthy life lost per 100 person-years. At population level (that is, spread between those affected and those not), migraine imposed the greatest burden in these young people: 0.13 years lost per 100 person-years. Again, this may not appear to be high. All of these symptom burdens, however, are on increasing trends with age, while population-level disability is additionally driven by increasing prevalences towards adult values of all headache types except UdH [19]. Adolescents are therefore more burdened than children, while pMOH is egregious among the headache types in its individual impact.

We noted that medication days were fewer than headache days, although only negligibly for pMOH. These age groups do not medicate all headaches, a reflection, presumably, of the short duration of most headaches, a well-recognised characteristic of these age groups. Of course, pMOH stands out: highly frequent, longer lasting and more intense, coupled with symptomatic medication intake on more days than not. The prevalence of pMOH was low overall (0.8% [1]), but our earlier publication revealed a rapid rise with age: from 0.3% in children to 1.3% in adolescents [1], headed towards the 3.2% found in our adult population-based study in Lithuania [19]. We noted in that study that “whatever causes there may be for highly frequent headache, there is a suspicion that medication overuse is, at least in part, a behaviour learned by children and adolescents from parents”. Here we see the increasing symptomatic burden with age of the episodic headaches – expressed in frequency, duration and therefore pTIS – as a plausible driver of increasing medication use. And concern is raised by the 3.1% with other headache on ≥15 days/month, who already use medication once a week on average.

We should note that, more than in adults, duration (a factor in pTIS and symptom-based disability estimates) may be underestimated because of intervening sleep – a commonly effective therapy. While this may reduce symptom burden, its consequences are reflected in activity indices. Indeed, although symptom burdens appeared modest, the reported consequential burdens tell a somewhat different story. Particularly among these is lost school time, with its likely impact on education: a mean of 0.5 days/4 weeks (2.5%) is not inconsiderable. For those with migraine it was 3.5%, while for pMOH it was 25%, which could only be highly damaging to education and prospects. Although the small numbers with pMOH call for cautious interpretation, the larger group with other headache on ≥15 days/month lost 1.2 days (6.0%), which is still a serious impact. We note here that estimates of lost school time based on HY, presumed to be free from recall error (but not necessarily from embellishment), generally paint a picture of rather greater severity. For all episodic headaches, actual lost days yesterday were always somewhat higher (overall, by about a third) than predicted from 4-week recall. That this was not so for headache on ≥15 days/month suggests, as might be expected, that established patterns of frequent headache and absences are more reliably recalled. The point, however, is that, if the HY data are true, the 2.5% based on recall for all headache may underestimate true absences of 3.3%, and the 3.5% for migraine may in reality be 4.7%. Furthermore, school absences were more frequent among adolescents, in whom the consequences would be more adverse, than in children.

On the issue of possible embellishment, we noted previously that HY appeared to be over-reported, according to predictions based on headache frequencies [1]. It is of interest, therefore, that actual lost days – presumably an objective observation – in those (subjectively) reporting HY were higher than expected rather than lower. This suggests that reports of HY in these age groups might not be in error when in conflict with recollections over 4 weeks. Our earlier scepticism [1] may have been at least partly misplaced.

For all headache types, more days were reported with limited activity than lost from school. This could be anticipated for two reasons. First, a day of limited activity does not imply a whole day lost (as is the case with lost school days). Second, “activity” embraces a wide range, from chores to socialising and play, much of which, unlike school, is essentially optional and may be abandoned without consequence. Even so, the 1.5 days/4 weeks (5.4%) reportedly affected in this way in those with migraine suggests a considerable life diminution, while the 8.4 days/4 weeks (almost 1 day in every three) reported by those with pMOH, and the 4.6 days (one in six) reported by the larger group with other headache on ≥15 days/month, are indicative of major life impairments.

Parental work was also a casualty, with one in 30 parents (3.3%) of children or adolescents with headache reportedly missing work as a consequence at least once in the preceding 4 weeks. We did not enquire further because the young participants were unlikely to be fully aware of how much time their parents lost, so the range might be wide, from an hour or two to multiple days. Furthermore, while this finding was obviously influenced by how many parents were actually in paid work, which was unknown for the same reason, it remains valid as a measure of burden at population level without this knowledge. Notably, parental work losses were strongly influenced by headache type, as demonstrated by RRs (with reference to UdH) of 2.0 for TTH, 6.9 for migraine and 16.7 for pMOH. Equally notably, despite the greater burdens among adolescents, including more days lost from school, it was parents of children rather than of adolescents who bore the brunt of these losses.

These various burdens were reflected in emotional impact scores. The six questions contributing to this score related principally to concentration, mood, fear of headache and coping with it, so the gradient observed – pMOH having greatest impact, followed by other headache on ≥15 days/month, migraine and TTH, and UdH having significantly the least – was as expected. QoL scores showed a similar picture, with the lack of impact attributable to UdH distinguishing it clearly from all other headache types, a finding that supports its recognition as a distinct entity. Incidentally, QoL scores seemed generally low: < 72% of the possible score of 36 even for those with no headache, while the validation of the full KINDL scale suggests “healthy” scores of about 80% [20]. However, we cannot draw conclusions from this since we used only selected KINDL questions that seemed relevant to headache, and there are, as yet, no comparable data from elsewhere.

Finally, we might note that, while UdH is not reported in any of these measures as a burdensome headache, which is unsurprising since, by definition, it is mild and short-lasting (< 1 h), it remains important firstly as a common headache in these age groups, accounting for one third of all reported headache (38.0% in children declining to 27.4% in adolescents) [1]) and secondly because it is believed to represent immature and presumably evolving forms of migraine and TTH [2]. It is this evolution, rather than any overall increase in headache prevalence, that appears to set the course between childhood and adult headache. The importance of this observation is in the question it raises: can this evolution be modified?

There are no similar data yet available from other countries, but these will be forthcoming for comparison as the Global Campaign completes its programme of schools-based studies around the world [6, 10].

The strengths and limitations of this study are as noted previously [1]: its tested and validated methodology [2, 6, 13] and adequate sample size [21], but a non-participating proportion of almost one third (32.6%). The last introduced a gender-bias (non-participation was higher among males), which could be dealt with by statistical correction [1], but possibly, also, others unknown. The principal cause – prior parental consent was not forthcoming from almost one quarter (24.2%) of pupils [1] – is an obstacle to research in these age groups without easy solution.

## Conclusions

Headache, common in children and adolescents, is associated with symptom burdens that may not, for most, be onerous, but the consequential burdens, in particular lost school days, are not insubstantial. These findings are of importance not only to health policy but also to educational policy in Lithuania.

## Data Availability

The data are held on file at University of Mersin. Once analysis and publications are completed, they will be freely available for non-commercial purposes to any person requesting access in accordance with the general policy of the Global Campaign against Headache.

## Abbreviations

ANOVA: Analysis of variance
CI: Confidence interval
d/m: days per month
DW: Disability weight
GBD: Global Burden of Disease (Study)
HALT: Headache-Attributed Lost Time (index)
HARDSHIP: Headache-Attributed Restriction, Disability, Social Handicap and Impaired Participation (questionnaire)
HY: Headache yesterday
ICHD: International Classification of Headache Disorders
LTB: Lifting The Burden
MOH: Medication-overuse headache
pMOH: probable MOH
pTIS: proportion of time in ictal state
QoL: Quality of life
RR: Relative risk
SD: Standard devation
TTH: Tension-type headache
UdH: Undifferentiated headache
